# Exploratory Biological Correlates of Cognitive and Language Performance in Healthy Pubertal Children: A Retrospective Cross-Sectional Study

**DOI:** 10.3390/children13081101

**Published:** 2026-08-18

**Authors:** Merve Savaş, Senanur Kahraman Beğen, Ömer Okuyan, Hafize Uzun

**Affiliations:** 1 Department of Speech and Language Therapy, Faculty of Health Sciences, Istanbul Atlas University, 34403 Istanbul, Türkiye; senanur.kahraman@atlas.edu.tr; 2 Department of Pediatrics, Faculty of Medicine, Istanbul Atlas University, 34403 Istanbul, Türkiye; omer.okuyan@atlas.edu.tr; 3 Department of Medical Biochemistry, Faculty of Medicine, Istanbul Atlas University, 34403 Istanbul, Türkiye; hafize.uzun@atlas.edu.tr

**Keywords:** puberty, language development, cognitive performance, neurodevelopment, children

## Abstract

**Background/Objectives:** Adolescence is a dynamic neurodevelopmental period during which structured language performance may reflect ongoing biological reorganization processes. This study examined whether inflammatory, nutritional, and hormonal biomarkers were modestly associated with structured language task performance and cognitive performance in healthy pubertal children. **Methods:** A total of 156 healthy pubertal children (78 female, 78 male; mean age of 11.84 ± 2.98 years) meeting Tanner Stage ≥ 2 were included in this retrospective cross-sectional study. Language was assessed with the Test of Language Development-Primary: Fourth Edition (TOLD-P:4; Turkish adaptation) and cognition with the Wechsler Intelligence Scale for Children-Revised (WISC-R). Pan-Immune-Inflammation Value (PIV), Prognostic Nutritional Index (PNI), Neutrophil-to-Lymphocyte Ratio (NLR), vitamin B12, 25-OH vitamin D, ferritin, and TSH were included as biomarkers. Two-block hierarchical OLS regression and Spearman correlations with Benjamini–Hochberg FDR correction were used. **Results:** The biomarker block provided a statistically significant incremental contribution to the spoken language model (ΔR^2^ = 0.066, *p* = 0.041; full model R^2^ = 0.287). The primary test statistic was ΔR^2^. In exploratory coefficient-level analyses that were not corrected for multiple comparisons, the PIV (β = +0.17, *p* = 0.033) and TSH (β = −0.17, *p* = 0.031) coefficients reached nominal significance. After FDR correction, PIV was positively associated with WISC-R Verbal score (ρ = +0.24, p_FDR = 0.019) and TSH negatively with TOLD-P:4 Speaking (ρ = −0.22, p_FDR = 0.029). Biomarker contribution to WISC-R Full-Scale IQ was non-significant (ΔR^2^ = 0.049, *p* = 0.223). **Conclusions:** At the block level, the biomarker set contributed significantly to the structured spoken-language model but not to the Full-Scale IQ model; however, one of the two FDR-significant bivariate associations involved a verbal cognitive measure (PIV with WISC-R Verbal). Any language-versus-cognition contrast should therefore be interpreted cautiously. The modest effect sizes suggest these relationships are best interpreted within a developmental and neuroimmune framework. Longitudinal and multimodal studies are needed to replicate these findings. Because the TOLD-P:4/TODİL was applied beyond its validated age range (4; 0–8; 11) and its cross-age scoring could not be verified from the retrospective records, all language-based findings are exploratory and require confirmation with an age-validated instrument; the study’s confirmatory finding is the FDR-significant association of PIV with age-appropriate WISC-R Verbal performance. Because the TOLD-P:4/TODİL composite scores could not be verified as comparable across the sample’s age range, all TOLD-P:4-based results reported here are exploratory and hypothesis-generating; the confirmatory analyses rely on the age-appropriate WISC-R outcomes.

## 1. Introduction

Adolescence is a critical developmental period during which social interactions become increasingly complex, academic demands intensify, and the need for self-expression grows markedly stronger. During this period, language transcends its role as a basic communicative tool and assumes a central function in identity formation, maintenance of social relationships, emotional regulation, and academic participation. Notable advances are observed in syntactic complexity, abstract vocabulary use, pragmatic competence, and discourse organization; the ability to express thoughts coherently becomes an important determinant of social adaptation [1]. This linguistic maturation unfolds concurrently with the intensive neurodevelopmental reorganization that occurs in the brain during puberty. Synaptic reorganization and myelination in the prefrontal cortex and associated networks have been reported to influence executive functions, social cognition, and linguistic organization [2].

Structured language task performance is considered a complex developmental construct requiring the simultaneous integration of multiple neurocognitive systems. Speech production encompasses verbal organization, rapid lexical access, motor planning, temporal sequencing, and verbal–motor coordination [3,4]. Consequently, it depends not only on grammatical knowledge but also on processing speed, motor execution, and neurophysiological organization [5]. Given ongoing cortical maturation during puberty, structured language performance may represent a potentially sensitive developmental indicator.

Vitamins and micronutrients are essential components of neurobiological processes involved in brain development. Iron, B-group vitamins, and thyroid hormones have been associated with myelination, neurotransmitter synthesis, cortical organization, and psychomotor speed [6,7,8,9]. Micronutrient deficiencies during childhood and adolescence may be associated with attention, memory, and academic performance [10].

Sleep disturbances are also important determinants of cognitive and structured language task performance during adolescence. Disruptions in sleep duration and quality have been associated with attention, working memory, executive functions, and verbal fluency [11]. Insufficient sleep may affect inflammatory responses and exert indirect effects on cognitive performance.

The relationship between systemic inflammation and cognitive functions has been increasingly investigated. Studies in adult populations have shown that elevated inflammatory burden may adversely affect attention, memory, and executive functions [12,13]. However, the majority of the existing literature focuses on adult or clinical populations; the impact of physiological biomarker variation on language and cognitive performance in healthy pubertal individuals remains largely unclear. Studies examining the relationship between expressive language processes and peripheral immune, nutritional, and endocrine indicators are particularly limited. Given that neurodevelopmental maturation is actively ongoing during puberty, investigating whether physiological biological variation is associated with linguistic organization and cognitive performance is of considerable importance.

In recent years, composite biomarker indices have been developed to comprehensively assess peripheral inflammatory burden. The Pan-Immune-Inflammation Value (PIV) integrates neutrophil, platelet, monocyte, and lymphocyte components into a single index reflecting systemic immune-inflammatory burden [14]. The Prognostic Nutritional Index (PNI) evaluates serum albumin levels and lymphocyte count to provide information about nutritional status and immune capacity [15]. However, studies examining these indices in relation to cognitive or structured language performance in healthy pubertal individuals remain scarce.

Therefore, the present study examined the associations of peripheral inflammation, nutritional status, and hormonal indicators with language and cognitive performance in healthy pubertal children, investigating whether inflammatory indices, vitamin levels, serum protein markers, and thyroid function were associated with these outcomes beyond the contribution of age, sex, and BMI, while also descriptively characterizing sleep-related variability in the sample.

## 2. Materials and Methods

### 2.1. Participants

A total of 156 healthy pubertal children who presented to a university hospital, had complete language and cognitive assessments and biochemical data, and met Tanner Stage ≥ 2 were included in this retrospective cross-sectional study. In the retrospective records, pubertal status was documented as a threshold indicator (Tanner Stage ≥ 2 reached) rather than as a graded stage-by-stage classification; consequently, the full ordinal distribution of Tanner stages and the stage-by-sex breakdown could not be reconstructed. Inclusion criteria: pubertal age range, complete language and cognitive assessments, and available laboratory data. Exclusion criteria: neurodevelopmental disorders, neurological illness history, clinical endocrine disease, acute infection or inflammatory disease, and significant missing data. Because the study was based on retrospective hospital records, the term “healthy” refers to children without documented neurological, neurodevelopmental, endocrine, acute infectious, or chronic inflammatory disease according to available clinical records. Therefore, the sample should not be interpreted as an ideal community-based healthy population, but rather as a clinically healthy retrospective hospital-based sample after application of the exclusion criteria.

Pubertal status (Tanner stage) was assessed by the examining pediatrician during the original clinical evaluation and retrieved from the records. Children were identified from retrospective hospital records of outpatient pediatric assessments in which biochemical testing and developmental/language-cognitive evaluations were available within the clinical record.

Sample size was evaluated using G*Power 3.1. With f^2^ = 0.10, α = 0.05, 1 − β = 0.80, and 9 predictors, the present sample size of 156 was considered sufficient for the planned regression analyses.

### 2.2. Instruments

#### 2.2.1. Language Assessment

Structured language performance was assessed using the Test of Language Development–Primary: Fourth Edition (TOLD-P:4; Turkish adaptation, TODİL). The instrument comprises six core subtests and three supplemental subtests and yields six composite standard scores: Listening, Organizing, Speaking, Grammar, Semantics, and Spoken Language [1,16].

Although the Turkish adaptation of the TOLD-P:4 was standardized for children aged 4; 0–8; 11 years, it was used in the present retrospective study as a structured language performance measure rather than as a diagnostic instrument for age-normed classification across the entire pubertal age range. This decision was made because standardized Turkish language assessment instruments comprehensively covering adolescence and the pubertal period are limited. Accordingly, TOLD-P:4-derived scores were used to examine interindividual variation in structured language task performance and were not used to identify or classify language impairment.

The TOLD-P:4 outcomes used in the present analyses were composite standard scores rather than raw subtest scores. In accordance with the scoring structure of the instrument, each composite was derived by combining the scaled scores of specified core subtests. The Listening composite comprised Picture Vocabulary and Syntactic Understanding; the Organizing composite comprised Relational Vocabulary and Sentence Imitation; the Speaking composite comprised Oral Vocabulary and Morphological Completion; the Grammar composite comprised Syntactic Understanding, Sentence Imitation, and Morphological Completion; the Semantics composite comprised Picture Vocabulary, Relational Vocabulary, and Oral Vocabulary; and the Spoken Language composite comprised all six core subtests. The three supplemental subtests—Word Discrimination, Phonemic Analysis, and Articulation—were not included in the six composite outcomes analyzed in this study. The same composite-score format was available for all participants.

For participants above the validated normative age ceiling, the retrospective clinical records contained only the final, already-computed TOLD-P:4 composite standard scores. The records did not retain the underlying raw and scaled subtest scores or sufficient scoring documentation to determine which age-based normative conversion, including whether the oldest available normative table, had been applied to these participants. Consequently, a raw-score sensitivity analysis could not be performed. The composite scores were therefore analyzed only as continuous indicators of relative within-sample structured-language performance and were not interpreted diagnostically or according to age-based normative reference values.

Chronological age was included as a covariate in all regression models to partially account for developmental variability across the broad age range. However, this statistical adjustment does not establish the normative validity or measurement equivalence of the TOLD-P:4 composite scores across the full pubertal age range.

The TOLD-P:4/TODİL was administered by qualified speech and language therapists as part of the routine clinical evaluation.

#### 2.2.2. Cognitive Assessment

Cognitive performance was assessed with the Wechsler Intelligence Scale for Children-Revised (WISC-R). Verbal and Performance domain scores and Full-Scale IQ were used in the analyses [17,18].

WISC-R was used because it was the only cognitive assessment version consistently available for all participants in the retrospective records. The retrospective records covered assessments conducted between 2023 and 2025. To preserve measurement consistency and comparability across the sample, different WISC versions were not combined. In this study, the reported WISC-R Verbal and Performance scores are the sums of the age-scaled subtest scores within each domain and are therefore not deviation IQ indices; only the Full-Scale IQ is expressed on the standard-score metric (M = 100, SD = 15). Accordingly, Verbal and Performance values are interpreted only as relative within-domain performance indicators, and no Verbal–Performance IQ discrepancy is computed.

The WISC-R Performance domain reflects nonverbal, perceptual–organizational abilities—including visual–spatial reasoning, perceptual organization, visual–motor coordination, and nonverbal problem-solving—whereas the Verbal domain reflects verbal comprehension, acquired knowledge, and verbal reasoning. The WISC-R was administered by the hospital’s clinical psychologist as part of the routine clinical evaluation.

#### 2.2.3. Sleep Assessment

Sleep status was assessed with the Sleep Disturbance Scale for Children (SDSC), a standardized parent-reported scale covering initiating/maintaining sleep, breathing problems, nocturnal awakenings, parasomnias, excessive daytime sleepiness, and sleep–wake transition disorders [19,20].

### 2.3. Biochemical and Hematological Parameters

Biochemical and hematological variables were organized according to their analytical role. Albumin, total protein, free T3, and free T4 were reported descriptively. PIV, PNI, NLR, vitamin B12, 25-OH vitamin D, ferritin, and TSH were examined in the correlation analyses. The primary regression model included six pre-specified biomarkers: PIV, PNI, ferritin, vitamin B12, vitamin D, and TSH. NLR was examined descriptively and correlationally but was not included in the primary regression model to avoid redundancy with PIV and to preserve model parsimony.

The six biomarkers were selected a priori to represent complementary physiological systems with hypothesized links to neurodevelopment, and each was computed and interpreted with a consistent directional meaning. PIV is a composite index of systemic immune–inflammatory burden derived from neutrophil, monocyte, platelet, and lymphocyte counts, with higher values indicating a greater pro-inflammatory state, which has been associated with poorer cognitive and language outcomes. PNI, combining serum albumin and lymphocyte count, indexes nutritional–immune reserve, with lower values reflecting poorer status. Ferritin reflects iron stores, which are essential for myelination and monoaminergic neurotransmission. Vitamin B12 is a cofactor for myelin synthesis and neuronal metabolism. 25-OH vitamin D has neurotrophic and neuroimmune roles relevant to brain development. TSH indexes thyroid function, which is critical for brain maturation and language development, with higher values reflecting reduced thyroid hormone output. Selecting markers that span inflammatory, nutritional, hematological, and endocrine systems allowed the model to probe several biologically plausible pathways while remaining parsimonious.

Biochemical and hematological data were obtained retrospectively from the hospital information system. All laboratory measurements were performed in the same hospital laboratory using routine clinical procedures. Because of the retrospective design, fasting status, exact time of blood sampling, and the precise interval between blood sampling and language/cognitive assessment could not be standardized for all participants; this was therefore considered a methodological limitation.

The following indices were calculated: NLR = Neutrophil/Lymphocyte [21]; PNI = 10 × Albumin (g/dL) + 0.005 × Lymphocyte (/mm^3^) [15]; PIV = (Neutrophil × Monocyte × Platelet)/Lymphocyte [14].

### 2.4. Statistical Analysis

All analyses were performed using IBM SPSS Statistics version 26.0 (IBM Corp., Armonk, NY, USA). The TOLD-P:4 Spoken Language composite standard score, derived from all six core language subtests, was selected as the primary structured-language outcome [3,4]. A two-block hierarchical multiple linear regression model was constructed. Block 1 included age, sex, and BMI. Block 2 added clinically pre-specified biomarkers, including PIV, PNI, ferritin, vitamin B12, vitamin D, and TSH. Biomarker predictors were z-score standardized before entry into the regression models, whereas age and BMI were entered in their original metric units. The primary test statistic was ΔR^2^. Individual biomarker regression coefficients were not corrected for multiple comparisons and were therefore treated as exploratory. Spearman correlations were computed with Benjamini–Hochberg false discovery rate (FDR) correction for 63 tests. Regression model assumptions were verified prior to interpretation. The SDSC total score was descriptively reported to characterize sleep-related variability in the sample but was not entered into the primary regression models. Statistical significance was set at *p* < 0.05. Given the exploratory nature of the study and the relatively large number of tested associations, findings were interpreted cautiously with emphasis on effect sizes, confidence intervals, and FDR-adjusted results.

Prior to the regression analyses, the data were screened for univariate outliers using standardized (z) scores and for influential observations using standardized residuals and Cook’s distance. No observations reached conventional thresholds warranting exclusion, and to preserve the full clinically defined sample, all 156 participants were retained in the analyses.

Additional exploratory sensitivity analyses were conducted for the TOLD-P:4 Spoken Language outcome. To examine whether biomarker-language associations varied by age, Age × PIV and Age × TSH interaction terms were added to the primary regression framework after mean-centering age. In addition, SDSC total score was entered as an intermediate block before the biomarker block to evaluate whether sleep disturbance altered the observed biomarker-language pattern.

### 2.5. Ethical Statement

The study was conducted in accordance with the Declaration of Helsinki and approved by the Istanbul Atlas University Non-Interventional Scientific Research Ethics Committee (decision no: 41; meeting no: 01; date: 26 January 2026; document no: E-22686390-050.99-90346). The ethics approval and consent waiver covered the retrospective use of anonymized pediatric clinical records, including language/cognitive assessment data and laboratory parameters.

## 3. Results

### 3.1. Descriptive Statistics

A total of 156 healthy pubertal children (78 male [50.0%], 78 female [50.0%]) were included. The mean age was 11.84 years (SD = 2.98; range 8.17–16.17). Descriptive statistics are presented in Table 1 and Table 2. Reference ranges are provided only for descriptive orientation and were not used for diagnostic classification.

**Table 1:** Descriptive statistics for demographic and anthropometric variables.

Variable	*n*	M (SD)	Median	Min–Max
Age (years)	156	11.84 (2.98)	12.33	8.17–16.17
Height (cm)	156	152.96 (15.00)	156.00	115.00–177.00
Weight (kg)	156	47.62 (14.99)	48.00	20.00–79.00
BMI (kg/m^2^)	156	19.94 (4.36)	19.22	12.33–31.64
Sex: male	78	50.0%	-	-
Sex: female	78	50.0%	-	-

Note. M = mean; SD = standard deviation; BMI = body mass index. Minimum age (8.17 years) is consistent with the pubertal inclusion criterion (Tanner Stage ≥ 2; ≥8 years for females, ≥9 years for males).

**Table 2:** Descriptive statistics for independent and dependent variables.

Variable	n	M (SD)	Median	IQR	Min	Max
PIV	156	443.56 (190.23)	369.14	216.88	168.74	958.36
PNI	156	61.82 (5.73)	61.30	7.28	51.80	73.75
NLR	156	1.63 (1.34)	1.31	0.53	0.65	8.82
Vit. B12 (ng/L)	156	299.44 (97.18)	296.00	120.00	24.00	568.00
Vit. D (ng/mL)	156	13.88 (7.61)	11.90	7.95	4.70	40.50
Ferritin (µg/L)	156	34.04 (23.26)	27.80	25.25	7.50	120.50
Albumin (g/dL)	156	4.76 (0.29)	4.70	0.30	4.10	5.70
Total protein	156	7.67 (0.36)	7.70	0.40	6.80	8.50
TSH (mU/L)	156	1.97 (0.86)	1.71	1.09	0.68	4.23
fT4 (ng/dL)	156	1.02 (0.09)	1.02	0.08	0.82	1.28
fT3 (ng/L)	156	3.51 (0.38)	3.51	0.58	2.82	4.25
SDSC Total	156	41.59 (8.87)	40.50	13.75	28.00	64.00
TOLD-P:4 Spoken	156	100.26 (13.20)	101.50	15.75	66.00	123.00
TOLD-P:4 Listening	156	97.43 (15.31)	100.00	12.75	24.00	121.00
TOLD-P:4 Organizing	156	105.20 (18.20)	106.00	21.00	22.00	130.00
TOLD-P:4 Speaking	156	96.78 (16.90)	103.00	18.00	22.00	118.00
TOLD-P:4 Grammar	156	99.61 (15.00)	104.00	11.00	37.00	121.00
TOLD-P:4 Semantics	156	99.43 (16.58)	99.50	16.50	31.00	123.00
WISC-R Verbal	156	47.13 (11.30)	47.00	14.75	21.00	74.00
WISC-R Perf.	156	46.35 (9.96)	47.50	17.25	27.00	66.00
WISC-R FS IQ	156	93.46 (16.31)	95.50	20.75	52.00	140.00

Note. M = mean; SD = standard deviation; IQR = interquartile range. PIV = (Neutrophil × Monocyte × Platelet)/Lymphocyte; PNI = 10 × Albumin + 0.005 × Lymphocyte; NLR = Neutrophil/Lymphocyte. Vitamin D in ng/mL (≥20 = adequate). TOLD-P:4 = Test of Language Development-Primary: Fourth Edition (Turkish adaptation); WISC-R = Wechsler Intelligence Scale for Children-Revised; SDSC = Sleep Disturbance Scale for Children; and FS = full scale. TOLD-P:4 values are composite standard scores derived from specified combinations of core subtest scores. Because the validated normative age range of the Turkish adaptation does not cover most of the present sample, these scores are analyzed only as continuous indicators of relative within-sample structured-language performance and are not interpreted diagnostically or according to age-based normative reference values.

### 3.2. Correlation Analysis

Spearman rank correlations were computed between seven biomarkers and nine cognitive and language outcomes (63 tests; Benjamini–Hochberg FDR correction). After FDR correction, two associations retained significance: PIV with WISC-R Verbal score (ρ = +0.24, *p* = 0.002, p_FDR = 0.019) and TSH with the TOLD-P:4 Speaking composite (ρ = −0.22, *p* = 0.006, p_FDR = 0.029). Notably, one of these two FDR-significant associations (PIV with WISC-R Verbal) involved a cognitive rather than a language measure. All other correlations did not reach significance (all p_FDR > 0.05). The complete correlation matrix is presented in Table 3.

### 3.3. Regression Analysis: TOLD-P:4 Spoken Language

**Figure 1:**
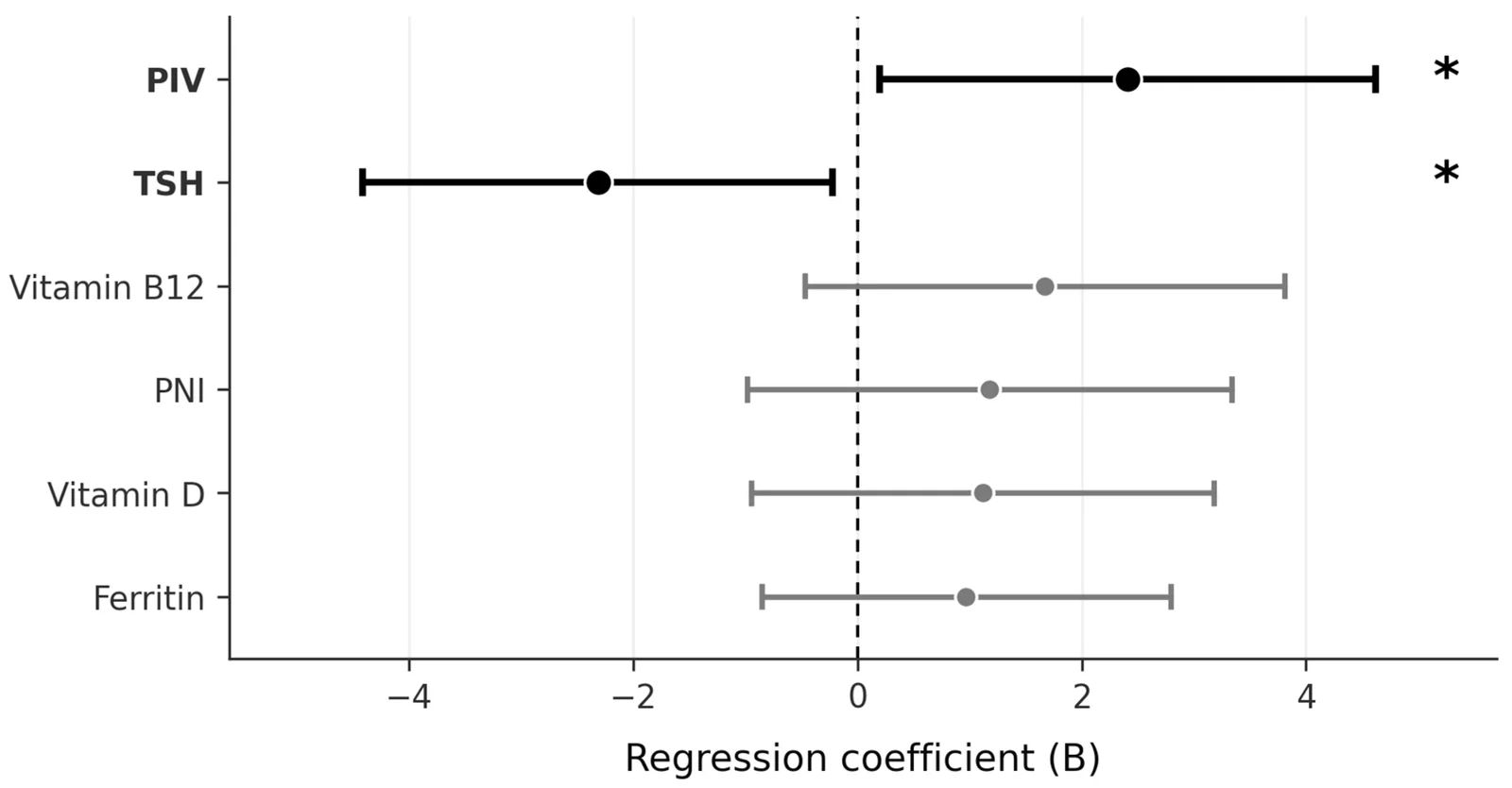
Regression coefficients (B, 95% CI) for the association between biomarkers and the TOLD-P:4 Spoken Language composite. Points indicate unstandardized regression coefficients (change in the composite per 1 SD increase in each biomarker); horizontal bars represent 95% confidence intervals. PIV and TSH reached significance (intervals excluding zero) and are shown in bold. Associations are exploratory and were not corrected for multiple comparisons. * *p* < 0.05.

A two-block hierarchical OLS regression was conducted on the TOLD-P:4 Spoken Language Score (n = 156). Model 1 was significant [R^2^ = 0.221, F(3, 152) = 14.37, *p* < 0.001, adj. R^2^ = 0.206]; age was a significant predictor (β = 0.31, *p* < 0.001). Block 2 yielded a significant incremental contribution [ΔR^2^ = 0.066, ΔF(6, 146) = 2.25, *p* = 0.041], full model R^2^ = 0.287 (adj. R^2^ = 0.243). The primary test statistic was ΔR^2^. In exploratory coefficient-level analyses that were not corrected for multiple comparisons, PIV (β = +0.17, *p* = 0.033) and TSH (β = −0.17, *p* = 0.031) were the individual predictors reaching significance (Figure 1) and should be interpreted as exploratory. Full results are in Table 4.

Regression diagnostics indicated no problematic multicollinearity, with all VIF values below 1.34. Visual inspection of residual plots supported linearity and homoscedasticity. No influential observations exceeding conventional Cook’s distance thresholds were detected. Sex was included as a covariate (Block 1) in both regression models; the sex coefficient was non-significant in the spoken-language model (β = −0.07, *p* = 0.292) and in the Full-Scale IQ model (β = −0.05, *p* = 0.457), indicating no independent sex effect on the outcomes after adjustment. Given the number of predictors relative to the sample size, sex-stratified models and sex × biomarker interaction tests were not pursued, as they would substantially reduce statistical power; this is noted as a limitation.

### 3.4. Regression Analysis: WISC-R Full-Scale IQ

The same two-block regression applied to WISC-R Full-Scale IQ (n = 156) yielded a significant Model 1 [R^2^ = 0.092, F(3, 152) = 5.13, *p* = 0.002, adj. R^2^ = 0.074]; age was a significant predictor (β = 0.28, *p* = 0.001). The biomarker block did not provide a significant incremental contribution [ΔR^2^ = 0.049, ΔF(6, 146) = 1.39, *p* = 0.223]. The Verbal-Performance IQ difference was non-significant [W = 1550, *p* = 0.719, r = 0.09]. Full results are in Table 5.

### 3.5. Exploratory Moderation and Sensitivity Analyses

In response to reviewer concerns regarding age-related variability and sleep status, additional exploratory moderation and sensitivity analyses were performed using the available complete-case dataset. Age × PIV and Age × TSH interaction terms were tested to determine whether the associations between biomarkers and spoken language differed across age. In addition, SDSC total score was included as an intermediate block to examine whether sleep disturbance altered the biomarker-language pattern. These supplementary analyses were not intended to replace the primary regression model but to evaluate the robustness of the observed findings. Results are summarized in Table 6.

In the age moderation model, the baseline covariate model including age, sex, and BMI was significant and explained 22.1% of the variance in TOLD-P:4 Spoken Language performance. Adding the biomarker block significantly increased the explained variance to 28.7%. The addition of Age × PIV and Age × TSH interaction terms produced only a small increase in explained variance and did not significantly improve model fit. In the SDSC sensitivity model, adding SDSC total score did not improve the model. Importantly, the biomarker block remained significant after SDSC total score was included, suggesting that the observed biomarker-language association was not primarily accounted for by sleep-related variability.

## 4. Discussion

This study examined whether inflammatory, nutritional, and hormonal biomarkers were associated with language and cognitive performance in healthy pubertal children. The findings suggest that biomarkers may be modestly but differentially associated with structured language performance compared to general cognitive performance. However, these findings should be interpreted within an exploratory and hypothesis-generating framework given the modest effect sizes and cross-sectional design. The statistically significant yet limited incremental contribution of the biomarker block to the spoken language model, which was not replicated in the general cognitive model, is particularly noteworthy. PIV showed a positive association with verbal cognitive performance, and TSH showed a negative association with speaking performance. Observed effect sizes were small; findings are more appropriately interpreted within a developmental and neurobiological context.

Two neurodevelopmental frameworks are important for interpreting these findings. First, adolescence is not a completed developmental stage but a dynamic period in which structural and functional reorganization continues, including synaptic reorganization, white matter maturation, and network integration in prefrontal and temporal regions [22]. Structured language performance during puberty may therefore reflect not only acquired capacity but also ongoing neurodevelopmental organization. The stronger appearance of biomarker associations in expressive language components suggests that linguistic organization may constitute a more sensitive developmental phenotype to these reorganization processes.

Second, the recent neuroimmunology literature demonstrates that the immune system plays an active role during normal brain development. Microglial activity and cytokine signaling contribute to synaptic pruning, neural network organization, and experience-dependent plasticity [23,24,25]. Low-level peripheral immune variation in healthy pubertal individuals may therefore carry different neurodevelopmental implications than pathological inflammation in adult clinical populations.

The direction of the positive PIV-verbal performance association is consistent with this developmental interpretation. In adult populations, systemic inflammation is associated with cognitive decline [12,13]. However, the present sample consisted of individuals without documented inflammatory or endocrine disease. Individual laboratory values were not necessarily within optimal or reference ranges: mean vitamin D was low, and PIV does not have a simple, universally accepted pediatric reference range. The developmental context may alter the direction and magnitude of immune-cognition relationships.

The negative TSH-speaking performance association is biologically plausible given thyroid hormones’ roles in myelination, psychomotor speed, and cortical organization [8,9]. Given that speech production encompasses motor planning, processing speed, and verbal–motor coordination, variation in the thyroid axis may be particularly relevant to expressive language. Because free T3 and free T4 were not included in the primary regression framework, the TSH finding should be interpreted cautiously and requires replication in studies with more detailed thyroid-axis modeling.

The more limited biomarker associations on general cognitive performance may be attributed to the greater stability and breadth of Full-Scale IQ. Age emerged as the strongest predictor in both models, underscoring the predominant role of maturational processes during puberty. Age, sex, and BMI were included as covariates; therefore, the observed biomarker associations were not merely attributable to basic demographic or anthropometric factors. Sleep status was descriptively characterized but was not included in the primary regression models. Future studies should examine sleep quality as a potential moderator or mediator of biomarker-language relationships [11]. Age was the strongest predictor of structured spoken language performance, which is expected given the broad developmental range covered by puberty. Importantly, the biomarker block remained significant after age, sex, and BMI were controlled, indicating that the observed associations were not merely attributable to age-related developmental differences.

The significant association with chronological age may reflect genuine maturational variability, limitations in the derivation of scores beyond the validated normative range, or both. Chronological age was therefore included as a covariate to reduce age-related confounding. However, this adjustment does not resolve the measurement-validity limitation or establish measurement equivalence of the TOLD-P:4 composite standard scores across the full pubertal age range.

Additional exploratory analyses further supported a cautious interpretation of the findings. Age did not significantly moderate the associations of PIV or TSH with spoken language performance, suggesting that these associations were not strongly age-dependent within the available dataset. Moreover, SDSC total score did not meaningfully alter the direction of the PIV and TSH coefficients. These findings suggest that sleep-related variability did not primarily account for the observed biomarker-language pattern; however, these analyses should be interpreted as exploratory given the secondary nature of the analyses.

Several limitations should be noted. The cross-sectional design precludes causal inference. The range of biomarker variation may have been limited given the clinically healthy sample. However, suboptimal vitamin D levels were prevalent across the cohort, which may reflect population-level nutritional characteristics rather than ideal physiological status. Therefore, the term ‘healthy’ should be interpreted as the absence of clinically diagnosed neurological, endocrine, or inflammatory disease rather than fully optimized nutritional status. Peripheral biomarkers do not directly reflect central neuroinflammatory processes; cytokine levels, neuroimaging, and neurophysiological measurements were not assessed. The absence of objective motor speech measures limits interpretation of speaking performance. Although several associations reached statistical significance, observed effect sizes were modest. Given the modest effect sizes, the observed associations should be interpreted cautiously, as residual confounding and unmeasured developmental or environmental influences cannot be excluded. In addition, potentially important environmental and socioeconomic variables-including parental education, socioeconomic status, language exposure, and academic environment-were not systematically assessed. Because pubertal status was recorded only as a threshold criterion (Tanner Stage ≥ 2) rather than as graded stages, stage-level distribution and stage-stratified or stage-adjusted analyses were not possible; chronological age served as the developmental proxy in all models.

Because parental education and household income are known to substantially influence language and cognitive performance, the absence of these socioeconomic measures is acknowledged as a limitation, and future studies should incorporate direct measures of parental education and income.

In addition, because of the retrospective design, fasting status, exact blood-sampling time, and the interval between blood sampling and the language/cognitive assessments could not be standardized, which may have introduced measurement variability.

A central measurement limitation concerns the use of TOLD-P:4 composite standard scores beyond the validated normative age range of the Turkish adaptation (4; 0–8; 11 years). Although the composites were constructed from specified combinations of scaled core-subtest scores, the retrospective records contained only the final composite scores and did not retain the underlying raw and scaled subtest scores or sufficient documentation to verify the normative conversion applied to older participants. Consequently, a raw-score sensitivity analysis could not be conducted, and the scores cannot be assumed to be normatively equivalent across the full age range. They were therefore interpreted only as relative, within-sample indicators of structured-language task performance. Future studies should use language assessments validated across adolescence.

## 5. Conclusions

Peripheral inflammatory and hormonal measures showed a modest, FDR-significant association with age-appropriate WISC-R Verbal performance in clinically healthy pubertal children, which we regard as the study’s primary robust finding; associations with the TOLD-P:4/TODİL language measure are reported as exploratory only, because that instrument was used beyond its validated age range and the comparability of its scores across the sample could not be verified, and they require confirmation with an age-validated instrument. Given the exploratory design, modest effect sizes, and retrospective hospital-based sample, these findings should be interpreted as preliminary and hypothesis-generating. Longitudinal, multimodal, and larger-sample studies are needed to replicate and extend these findings.

## Figures and Tables

**Table 3:** Spearman correlations between biomarkers and cognitive and language scores.

Variable	TOLD-P:4 List.	TOLD-P:4 Org.	TOLD-P:4 Speak.	TOLD-P:4 Gram.	TOLD-P:4 Sem.	TOLD-P:4 Spoken	WISC-R Verb.	WISC-R Perf.	WISC-R FS
PIV	+0.14	+0.10	+0.05	+0.11	+0.16	+0.17 *	† +0.24 **	−0.06	+0.21 *
PNI	+0.08	+0.05	+0.02	+0.04	−0.03	+0.04	+0.12	+0.15	+0.17
NLR	+0.03	+0.15	−0.11	+0.05	+0.04	+0.07	+0.08	−0.05	+0.08
TSH	+0.07	−0.12	† −0.22 **	−0.17	−0.09	−0.17 *	−0.11	−0.09	−0.13
Vit. B12	+0.12	+0.08	+0.04	+0.13	+0.10	+0.13	+0.14	+0.09	+0.13
Vit. D	+0.09	+0.06	+0.03	+0.08	+0.07	+0.08	+0.10	+0.07	+0.10
Ferritin	+0.07	+0.09	+0.06	+0.10	+0.08	+0.09	+0.12	+0.08	+0.11

Note. Benjamini–Hochberg FDR correction applied. † Significant after FDR correction: PIV × WISC-R Verbal (p_FDR = 0.019); TSH × TOLD-P:4 Speaking (p_FDR = 0.029). List. = listening; Org. = organizing; Speak. = speaking; Gram. = grammar; Sem. = semantics; and FS = full scale. * *p* < 0.05; ** *p* < 0.01 (uncorrected).

**Table 4:** Hierarchical multiple linear regression model for TOLD-P:4 Spoken Language composite score (n = 156).

Variable	B	SE	β	t	*p*	95% CI	VIF
Model 1—Covariates R^2^ = 0.221, Adj. R^2^ = 0.206							
Age (years)	1.24	0.31	0.31	4.01	<0.001	[0.63, 1.85]	1.12
Sex (female = 1)	−0.89	0.86	−0.07	−1.03	0.304	[−2.59, 0.81]	1.04
BMI (kg/m^2^)	−0.24	0.19	−0.09	−1.26	0.210	[−0.62, 0.14]	1.09
ΔR^2^ = 0.066, Full model: R^2^ = 0.287, Adj. R^2^ = 0.243							
Age (years)	1.18	0.32	0.30	3.71	<0.001	[0.55, 1.81]	1.18
Sex (female = 1)	−0.92	0.87	−0.07	−1.06	0.292	[−2.64, 0.80]	1.06
BMI (kg/m^2^)	−0.21	0.20	−0.08	−1.09	0.279	[−0.60, 0.17]	1.14
PIV (z)	2.41	1.12	+0.17	2.15	0.033	[0.20, 4.62]	1.21
PNI (z)	1.18	1.09	0.08	1.08	0.282	[−0.98, 3.34]	1.34
Ferritin (z)	0.97	0.92	0.07	1.05	0.294	[−0.85, 2.79]	1.19
Vit. B12 (z)	1.67	1.08	0.12	1.55	0.124	[−0.47, 3.81]	1.27
Vit. D (z)	1.12	1.04	0.08	1.07	0.286	[−0.95, 3.18]	1.23
TSH (z)	−2.31	1.06	−0.17	−2.18	0.031	[−4.41,−0.22]	1.18

Note. Block 1: demographic and anthropometric covariates (age, sex, BMI). Block 2: six pre-specified biomarkers. Biomarker predictors are z-score standardized; therefore, B values for biomarkers represent the expected change in the outcome per 1 SD increase in the biomarker. Age and BMI are entered in their original metric units. ΔR^2^ test: F(6, 146) = 2.25, *p* = 0.041. Intercept terms are not displayed to improve table readability. B = unstandardized coefficient; SE = standard error; β = standardized coefficient; CI = confidence interval; VIF = variance inflation factor.

**Table 5:** Hierarchical multiple linear regression model for WISC-R Full-Scale IQ (n = 156).

Variable	B	SE	β	t	*p*	95% CI	VIF
Model 1—Covariates R^2^ = 0.092 **, Adj. R^2^ = 0.074							
Age (years)	1.89	0.57	0.28	3.30	0.001	[0.76, 3.02]	1.12
Sex (female = 1)	−1.24	1.56	−0.06	−0.80	0.428	[−4.32, 1.84]	1.04
BMI (kg/m^2^)	−0.31	0.35	−0.07	−0.89	0.375	[−1.00, 0.38]	1.09
ΔR^2^ = 0.049 (n.s.) Full model: R^2^ = 0.141, Adj. R^2^ = 0.088							
Age (years)	1.82	0.58	0.27	3.14	0.002	[0.67, 2.97]	1.18
Sex (female = 1)	−1.18	1.58	−0.05	−0.75	0.457	[−4.30, 1.94]	1.06
BMI (kg/m^2^)	−0.28	0.36	−0.06	−0.79	0.433	[−0.99, 0.43]	1.14
PIV (z)	2.14	2.03	0.09	1.05	0.294	[−1.88, 6.16]	1.21
PNI (z)	1.42	1.97	0.06	0.72	0.473	[−2.48, 5.32]	1.34
Ferritin (z)	0.87	1.67	0.04	0.52	0.603	[−2.43, 4.17]	1.19
Vit. B12 (z)	1.95	1.96	0.08	1.00	0.320	[−1.92, 5.82]	1.27
Vit. D (z)	1.38	1.88	0.06	0.73	0.464	[−2.35, 5.11]	1.23
TSH (z)	−1.84	1.92	−0.08	−0.96	0.339	[−5.64, 1.96]	1.18

Note. Model structure identical to Table 4. Biomarker predictors are z-score standardized; age and BMI are entered in their original metric units. Intercept terms are not displayed to improve table readability. Biomarker block (Model 2) does not provide significant incremental contribution. ΔR^2^ test: F(6, 146) = 1.39, *p* = 0.223. n.s. = not statistically significant. ** *p* < 0.01.

**Table 6:** Summary of additional moderation and sensitivity analyses for TOLD-P:4 Spoken Language.

Analysis	Step/Block	n	R^2^	Adj. R^2^	F or ΔF	*p*
Age moderation	Block 1: age, sex, BMI	156	0.221	0.206	F = 14.37	<0.001
	Block 2: + PIV, PNI, ferritin, B12, vitamin D, TSH	156	0.287	0.243	ΔF = 2.25	0.041
	Block 3: + Age × PIV and Age × TSH	156	0.293	0.239	ΔF = 0.630	0.534
SDSC sensitivity	Block 1: age, sex, BMI	156	0.221	0.206	F = 14.37	<0.001
	Block 2: +SDSC total	156	0.221	0.201	ΔF = 0.065	0.799
	Block 3: + PIV, PNI, ferritin, B12, vitamin D, TSH	156	0.288	0.239	ΔF = 2.25	0.042

Note. BMI = body mass index; SDSC = Sleep Disturbance Scale for Children; PIV = Pan-Immune-Inflammation Value; PNI = Prognostic Nutritional Index; and TSH = thyroid-stimulating hormone. Biomarkers are z-score standardized within the complete-case sample. In the moderation model, age is centered before creating interaction terms.

## Data Availability

Anonymized derived data supporting the findings of this study may be made available from the corresponding author upon reasonable request and subject to institutional and ethical approval. Individual-level clinical records cannot be shared publicly due to privacy restrictions related to pediatric retrospective health data.

## Abbreviations

The following abbreviations are used in this manuscript:

**Abbreviation**	**Expansion**
BMI	Body Mass Index
FDR	False Discovery Rate
NLR	Neutrophil-to-Lymphocyte Ratio
OLS	Ordinary Least Squares
PIV	Pan-Immune-Inflammation Value
PNI	Prognostic Nutritional Index
SDSC	Sleep Disturbance Scale for Children
SD	Standard Deviation
TOLD-P:4	Test of Language Development-Primary: Fourth Edition (Turkish adaptation: TODİL)
TSH	Thyroid-Stimulating Hormone
WISC-R	Wechsler Intelligence Scale for Children-Revised
